# DR-region of Na^+^/K^+^ ATPase is a target to treat excitotoxicity and stroke

**DOI:** 10.1038/s41419-018-1230-5

**Published:** 2018-12-18

**Authors:** Meimei Shi, Lei Cao, Xu Cao, Mengyuan Zhu, Xingzhou Zhang, Zhiyuan Wu, Siping Xiong, Zhizhong Xie, Yong Yang, Jingyu Chen, Peter T. H. Wong, Jin-Song Bian

**Affiliations:** 1 Department of Pharmacology, Yong Loo Lin School of Medicine, National University of Singapore, Singapore, 117600 Singapore; 2 State Key Laboratory of Natural Medicines, Jiangsu Key Laboratory of Drug Discovery for Metabolic Disease, Center for New Drug Safety Evaluation and Research, China Pharmaceutical University, Nanjing, 211198 China; 3 Lung Transplant Group, Wuxi People’s Hospital Affiliated to Nanjing Medical University, Wuxi, 214021 Jiangsu PR China; 4 National University of Singapore (Suzhou) Research Institute, Suzhou, 215123 China

## Abstract

Na^+^/K^+^ ATPase (NKA) is important in maintaining cellular functions. We found that loss of NKA activities in NKAα1^+/−^ mice is associated with increased susceptibility to ischemic injuries following transient middle cerebral artery occlusion (tMCAO). This is corroborated by the neuroprotective effects of an antibody raised against an extracellular DR region (^897^DVEDSYGQQWTYEQR^911^, sequence number as in rat) of NKAα subunit (DR-Ab) in both preventive and therapeutic settings. DR-Ab protects cortical neurons against glutamate-induced toxicity by stimulating activities of NKA and Na^+^/Ca^2+^ exchanger (NCX), which resulted in accelerated Ca^2+^ extrusion. DR-Ab also enhanced the association between NKA and GluR2 and therefore reduced the internalization of both proteins from membrane induced by glutamate toxicity. The mechanism appears to involve suppression of GluR2 phosphorylation through PKCα/PICK pathway. Our data indicate that DR-region of NKA may be a novel therapeutic target for drug development for the treatment of ischemic stroke.

## Introduction

Na^+^/K^+^ ATPase (NKA) is responsible for maintaining the electrochemical gradient, and hence the membrane potential, of the cell. The excitability of the neuronal membrane results directly from the fact that its resting potential is maintained in the range of −60–90 mV. NKA interacts functionally with the plasma membrane Na^+^/Ca^2+^ exchanger (NCX) to prevent Ca^2+^ overload^1^ and neuronal apoptosis in excitotoxic stress. Inhibition of NKA may induce excitotoxicity^2^. Moreover, NKA is enriched at synapses where it is associated with α-amino-3-hydroxy-5-methyl-4-isoxazolepropionic acid receptors (AMPARs), which is a driving force for excitation. NKA dysfunction can induce a rapid reduction in the expression of cell-surface AMPAR leading to a long-lasting depression in synaptic transmission^3^. Thus, NKA may be potentially an important target to counter excitotoxicity.

Following the onset of ischemia, rapid depletion of cellular ATP may impair NKA functions and induces the failure of the ATP-dependent glutamate re-uptake to produce an excessive accumulation of extracellular glutamate and the resultant Ca^2+^-overload through activation of N-methyl-D-aspartate receptors (NMDAR) and voltage-dependent calcium channels. However, the role of NKA in stroke has not been well studied.

The NKA inhibitor ouabain is known to cause necrotic cell death at micromolar concentrations^4^, but stimulates NKA activity at nanomolar concentrations and acts as a signal transducer by activating the Ras-Raf-MAPK-PI3K/Akt signaling cascade^5^. Such nanomolar ouabain-induced increase in NKA activity has been reported to be neuroprotective against excitotoxicity^6,7^. It was also reported that digoxin at 65 mg/kg stimulated NKA in vivo but not at 130 mg/kg and higher doses^7^. Several cardiac glycosides (neriifolin, ouabain, digitoxin, and digoxin), however, have been reported to produce neuroprotection at 3–30 µM concentrations^8^. In addition, neriifolin at 1.5 mg/kg significantly reduced infarct volume in rats subjected to transient middle cerebral artery occlusion (tMCAO). Unfortunately, NKA activities were not determined in this study^8^. There are other corroborating findings including stimulation of NKA protecting cells against hypoxia-reperfusion induced injury via the PI3K/AKT and ERK pathways^9^; natural products protecting against H_2_O_2_-induced cell death through enhancing NKA activity^10^; and hypoxic-postconditioning protecting against transient global cerebral ischemia in rats through preserving the activity of NKA in hippocampal CA1^11^. However, the cardiac glycosides do not seem to be the appropriate pharmacologic tools for NKA activation based on the loss of effects and potential toxicity at higher concentrations or doses.

We and another group previously reported that an antibody against the extracellular region ^897^DVEDSYGQQWTYEQR^911^ (DR region) of M7/ M8 (DR-Ab) on NKA α subunit stimulates NKA activities^12–14^. We also demonstrated that this DR-Ab may protect cardiac myocytes against ischemic injury^12^. Therefore, we consider it worthwhile to investigate if activation of NKA with DR-Ab may also protect ischemic injuries in the brain. We hypothesized that DR-Ab is neuroprotective against ischemic injuries by activating NKA and thus reducing excitotoxicity and calcium overload.

## Materials and methods

### Chemicals and reagents

DR peptide (DVEDSYGQQWTYEQR) was purchased from 1st Base, Singapore, which was used to raise the DR-Ab and also to block DR-Ab. PKCα antibody and phosphor-GluR2ser880 antibody were purchased from Cell Signalling (Danvers, MA 01923, USA). GluR2 antibody was purchased from Abcam. NKAα antibody (H-3, sc-48345), NKAα1 antibody (sc-21712), NKAα2 antibody (sc-31391), NKAα3 antibody (sc-58631), EAAT1 antibody (Cell Signaling Technology, #5684), EAAT2 antibody (Cell Signaling Technology, #3838), goat anti-rabbit, and goat anti-mouse secondary antibodies were purchased from Santa Cruz Biotechnology (Santa Cruz, CA95060, USA). Fura-2 AM, Fluo-4 AM, Alexa Fluor 568 conjugated goat anti-rabbit IgG (H+L), and Alexa Fluor 488 conjugated goat anti-mouse IgG (H+L) were from Invitrogen Corporation (Carlsbad, CA, USA).

### Antibody generation and purification

Rats were administered subcutaneously with KLH (keyhole limpet hemocyanin) conjugated DR peptide (^897^DVEDSYGQQWTYEQR^911^, sequence number as in rat) biweekly. The first dose was 200 µg protein emulsified with complete Freund’s adjuvant (CFA) followed by 3 doses of 100 µg protein emulsified with IFA (incomplete Freund’s adjuvant). The serum was collected 5 days after the last immunization. Serums were collected when their A_450_ values were higher than that of control serums by 2.1-fold. The DR-Ab was purified using protein A/G spin column (ThermoFisher, catalog# 89962) according to manufacturer’s instructions. Meanwhile, serum from unimmunized rat was also collected and similarly purified. The purified antibodies were dissolved in PBS and stored at −80 °C. DR-OVA was used to coat ELISA plates. The wells were then incubated with serial dilutions of anti-DR IgG, and the bound antibody was detected by the addition of peroxidase-conjugated goat anti-rat antibody followed by tetramethylbenzidine substrate. Absorption was detected at 450 nm.

### Transient middle cerebral artery occlusion (tMCAO) and stereotaxic injection of DR-Ab

**Fig. 1:**
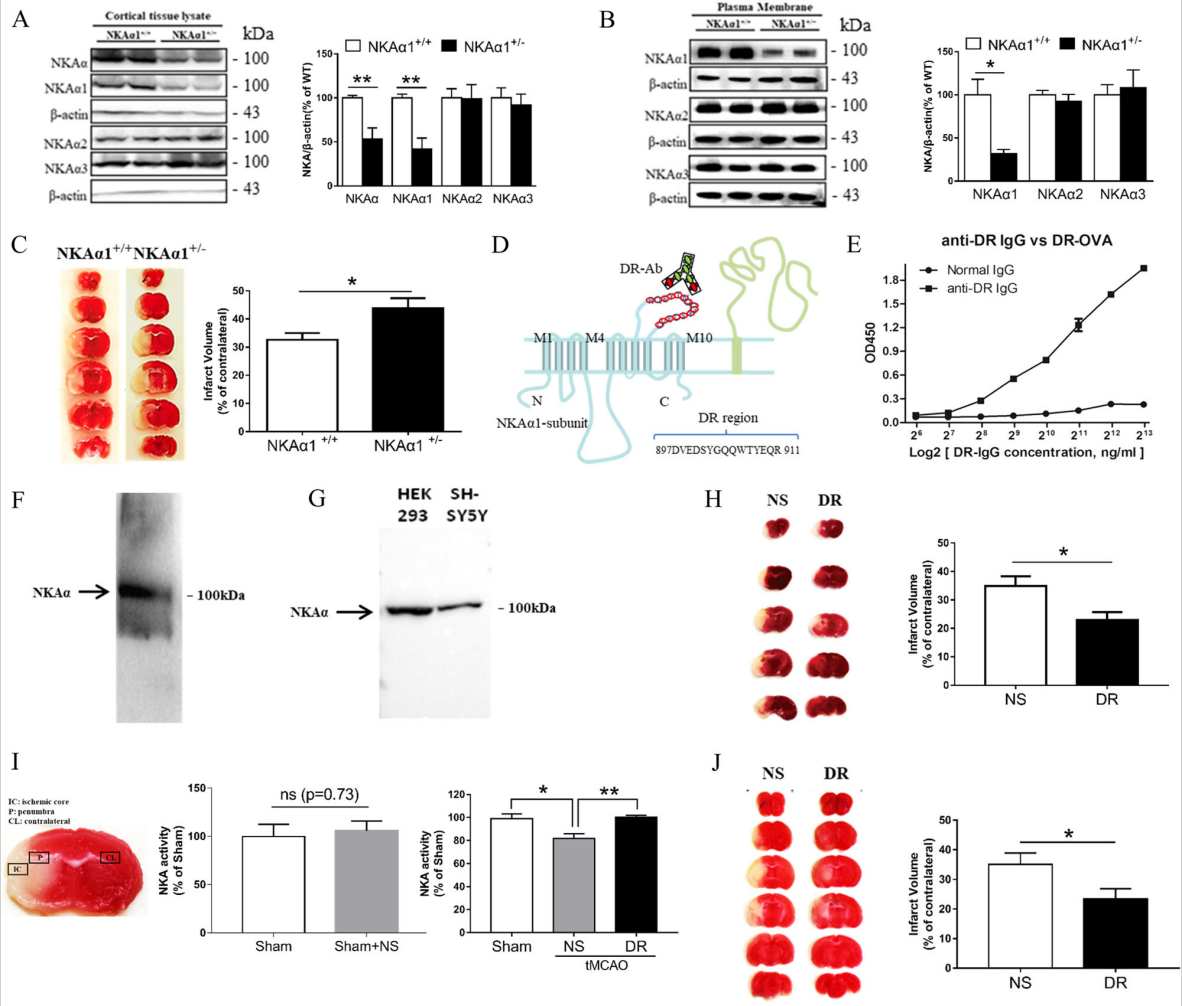
Infarct volumes in NKAα1^+/−^ and NKAα1^+/+^ mice after tMCAO and effect of DR-Ab on infarct volume in tMCAO mice. **a**, **b** Western blots showing that protein levels of cortical tissue lysate (**a**) and plasma membrane (**b**) of total NKAα (**a**) and NKAα1 were markedly reduced in NKAα1^+/−^ mice, but not NKAα2 and NKAα3. *n* = 4–5, Unpaired *t* test, two-tailed, *p< 0.05, **p < 0.01 vs NKAα1^+/+^ group. **c** Infarct volumes induced by tMCAO (60 min ischemia followed by reperfusion). TTC stained sections of a representative brain from each group. *n* = 12 for NKAα1^+/+^ group, *n* = 10 for NKAα1^+/−^ group, Unpaired *t* test, two-tailed, *p < 0.05 vs NKAα1^+/+^ group. **d**–**g** Generation of DR-Ab. **d** Schematic illustration showing the extracellular DR region of NKAα1subunit that binds DR-Ab. **e** ELISA of DR-Ab. **f**, **g** Representative western blots showing NKAα1 detected from membrane lysate in mouse brain (**f**), HEK293 and SH-SY5Y cells (**g**) using DR-Ab. **h** Infarct volumes induced by tMCAO (60 min ischemia followed by reperfusion). TTC stained sections of a representative brain from each group. DR, DR-Ab (30 µg/mouse*,* i.c.v.) pretreated group; NS, IgG purified from normal rat serum. *n* = 10 for NS group, *n* = 7 for DR group, Unpaired *t* test, two-tailed, *p < 0.05 vs NS group. **i** Left panel, illustration of penumbra area in tMCAO model; middle panel, NKA activity in sham and sham+NS group, *n* = 4/group, *t*-test; right panel, NKA activity in the penumbra area of the tMCAO mice. *n* = 5/group. One-way ANOVA followed by Bonferroni’s test, *p < 0.05, **p < 0.01. **j** Infarct volumes induced by tMCAO (60 min ischemia followed by reperfusion). TTC stained sections of a representative brain from each group. DR, DR-Ab (30 µg/mouse, i.c.v.) post-treated group; NS, IgG purified from normal rat serum. *n* = 9 for NS group, *n* = 11 for DR group, Unpaired *t* test, two-tailed, *p < 0.05 vs NS group

NKAα1^+/−^ mice were generated and kindly provided by Dr. Jerry B. Lingrel in the University of Cincinnati, USA, as a gift^15^. These mice were then bred in our animal facilities for use. Age-matched male NKAα1^+/+^ and NKAα1^+/−^C57/bl6 mice (8 weeks old) were subjected to tMCAO as described^16^. Briefly, anesthesia was induced by inhalation of 5% isoflurane, rectal temperature was maintained with a heating pad at 37 ℃ during the surgery, silicon coated monofilament (Doccol Corporation) was inserted into the internal carotid and advanced ~10 mm to occlude the origin of the MCA. Reperfusion was allowed after 60 min by withdrawing the filament. DR antibody (2 μl/mouse, 30 μg) or normal serum (2 μl/mouse) was randomly administrated 1 h before the tMCAO or 1 h after reperfusion by microinjection into the left lateral ventricle (1 mm ML, −0.45 mm AP from bregma 1.85 mm below the dura) as described^17^. The sham-operated animals were similarly treated but without MCA occlusion. All animals were placed in a single cage under a heating lamp to ensure the temperature (37 °C) was maintained during recovery for at least 2 h observation. After recovery, animals were returned to their cages with free access to food and water. Animals were killed 24 h after tMCAO. Fresh brain sections were stained using triphenyl tetrazolium chloride (TTC) to visualize the infarct regions. Infarct volume corrected for edema was calculated using Image-Pro Plus 5.0 according to the following equation: volume of contralateral hemisphere − volume of non-lesioned area in ipsilateral hemisphere)/volume of contralateral hemisphere × 100%. The investigators were blinded during sample allocation or result analysis. All animal procedures used in this study were conducted in strict compliance with the Institutional Animal Care and Use Committee of the National University of Singapore. For the experiment on evaluating the infarct volume of NKAα1^+/+^ and NKAα1^+/−^ mice in tMCAO model (Fig. 1c), 14 mice were used in NKAα1^+/+^ group, 2 mice were excluded because of incomplete occlusion, 12 mice were used in NKAα1^+/−^ group, 2 mice were excluded because of incomplete occlusion. For the experiment on evaluating the effect of pretreated DR-Ab on tMCAO model (Fig. 1h), 12 mice were used in NS group, 2 mice were excluded because of incomplete occlusion, 10 mice were used in DR-Ab group, 3 mice were excluded because of incomplete occlusion. While in post-treated DR-Ab experiment (Fig. 1j), 11 mice were used in NS group, 2 mice were excluded because of incomplete occlusion, 12 mice were used in DR-Ab group, 1 mouse were excluded because of incomplete occlusion.

### Ischemic core and penumbra dissection

The method of dissecting ischemic core and penumbra was described^18^. Briefly, in each animal the rostral side (2 mm) and caudal side (2 mm) of the brain was discarded. Regions from the right and left hemispheres of the remained part of the brain that corresponded to the ischemic core and penumbra were dissected. First, we identified the midline between the two hemispheres and then made a longitudinal cut from top to bottom ~1.5 mm from the midline through each hemisphere. Then we made a transverse diagonal cut at approximately the “1 o’clock” position to separate the core (i.e., striatum and overlying cortex) from the penumbra (adjacent ventrolateral neocortex).

### Preparation of primary cortical neurons

Primary cultured cortical neurons were prepared from E17 Sprague-Dawley rats or NKAα1^+/+^ and NKAα1^+/−^ mice as described before^19^. Briefly, the cerebral cortex was carefully dissected out and transferred in ice cold PBS and dissociated using trypsin (0.25%, 12 min at 37 °C) and a glass Pasteur pipette. Neurons were plated onto poly-d-lysine-treated glass cover slips or culture plates in DMEM supplemented with 10% FBS and 1% penicillin/streptomycin. The cells were incubated in a 5% CO_2_ incubator at 37 °C for 3–4 h, after which the media were replaced with serum-free Neurobasal/B27/glutamine media and maintained at 37 °C in a 5% CO_2_ incubator. Half of the medium was exchanged every 3 days. All experiments were performed on neurons that were cultured for 12–14 days in vitro (DIV).

### Cell viability and apoptosis assay

Cell viability was evaluated with the 3-(4,5-dimethylthiazol-2-yl)-2,5-diphenyltetrazolium bromide (MTT) assay as described previously with several modifications^20^. Briefly, neurons were pretreated with 0.15 mg/ml DR-Ab for 60 min and then incubated with different concentrations of glutamate (10, 100, 200 μM) for 24 h. One hundred microliters of fresh medium containing 0.5 mg/ml MTT was added and incubated at 37 ℃. After incubation for 4 h, the culture medium containing MTT was removed. Dimethyl sulfoxide (100 μl) was then added and the absorbance at 570 nm was measured using a spectrophotometric plate reader (Safire2, Tecan Group Ltd.).

To visualize nuclear morphology, cells were fixed in 4% paraformaldehyde and stained with DAPI. Uniformly stained nuclei were scored as healthy viable cells. Condensed or fragmented nuclei were scored as apoptotic. To obtain unbiased counting, Petri dishes were coded, and cells were scored blindly without knowledge of their prior treatment.

### Reactive oxygen species (ROS) measurement

The generation of reactive oxygen species was estimated using the dichlorofluorescin diacetate assay. Cells were incubated with 100 µM glutamate for different time scale (0.5, 1, 1.5, 2 h) in a 96-well culture plate with or without DR-Ab pretreatment for 1 h. After washing with PBS solution, cells were then incubated with 10 µM dichlorofluorescin diacetate (Sigma) for 30 min at 37 °C in phenol-free DMEM medium in the dark. The change in fluorescence of oxidized dichlorofluorescin was measured at excitation and emission wavelengths of 488 nm and 527 nm, respectively, under fluorescent microscope or using a fluorescence analyzer (Spectra Fluor Plus Tecan, Switzerland).

### Co-immunoprecipitation assay

Cortical neurons were lysed and equal amounts of protein were then incubated with anti-PICK1 antibody or anti-NKAα1 antibody overnight at 4 °C. Protein G-Agarose was added and incubated for another 4 h at room temperature the next day. The beads were washed three times with lysis buffer and mixed with 4 × loading buffer, boiled for 5 min at 95 °C and separated in 8% SDS-PAGE. The proteins were detected with western blots.

### Immunofluorescence labeling and protein colocalization

Cortical neurons were fixed in 4% formaldehyde and then permeabilized with 0.1% Triton X-100, followed by incubation with mouse anti-NKAα1 antibody or rabbit anti-GluR2 antibody overnight at 4 °C. After washing three times with PBS, cells were incubated with goat anti-mouse-Alexa 468 or goat anti-rabbit-Alexa 568 (Invitrogen, 1:400) for 2 h at room temperature before they were mounted with DAPI containing mounting medium (Invitrogen, Carlsbad, CA, USA). Photos were taken using a fluorescence microscope (Nikon, Japan).

### NKA activity assay

The penumbra area of fresh brain tissues were collected for NKA activity assay using a Fluorimetric SensoLyte FDP Protein Phosphatase Assay Kit (AnaSpec, Japan) according to manufacturer’s instructions. The enzyme activity of NKA was defined as the difference between the intensity of fluorescein in the presence and absence of 1 mM ouabain in the reaction mixture.

### Biotinylation of cell surface proteins

Surface proteins were labeled with EZ-Link SULFO-NHS-SS-biotin (1 mg/ml, Pierce Chemical Co., Rockford, IL, USA) for 1 h as described before^21^. Cells were then rinsed with PBS containing 100 mM glycine thoroughly to quench unreacted biotin and then lysed in modified radio-immuno-precipitation assay (RIPA) buffer (50 mM Tris-HCl, pH 8; 150 mM NaCl; 1% Triton X-100 and 1% sodium deoxycholate; 10 μg/ml leupeptin; 100 μg/ml TPCK; and 1 mM PMSF). Proteins (150–300 μg) were incubated overnight at 4 °C with end-over-end shaking in the presence of Streptavidin beads (Pierce Chemical Co.). Beads were thoroughly washed, resuspended in 30 μl loading buffer, and analyzed with Western blots.

### Plasma membrane protein isolation

Plasma membrane proteins were isolated using the plasma membrane protein extraction kit (ab65400, Abcam). The brain tissues were harvested and washed twice with ice cold PBS. These tissues were resuspended in homogenization buffer and lysed using a dounce homogenizer (50 strokes). The homogenate was centrifuged to obtain cytosol fraction in the supernatant. The pellet was further purified according to the manufacturer’s instructions to obtain purified plasma membrane proteins.

### Western blotting analysis

Protein concentrations were determined by the Lowry method. Protein samples were separated by 8–12% SDS-PAGE and transferred on to a nitrocellulose membrane. After blocking at room temperature in 10% milk in TBST buffer (10 mM Tris-HCl, 120 mM NaCl, and 0.1% Tween 20, pH 7.4) for 1 h, the membrane was probed with various primary antibodies at 4 °C overnight. Membranes were then washed three times in TBST, followed by incubation with 1:10,000 dilutions of horseradish peroxidase-conjugated anti-rabbit/mouse IgG at room temperature for 1 h and washed three times in TBST. Visualization was carried out using an enhanced chemiluminescence kit (GE Healthcare). The density of the bands was quantified by densitometry analysis of the scanned blots using ImageJ.

### Intracellular calcium measurement

Cultured neurons were incubated with 2 μM fura-2/AM or 10 μM fluo-4/AM for 30 min as we described^22^. The unincorporated dye was removed by washing the cells twice in fresh incubation solution. Loaded cells were maintained at room temperature for 30 min before measurement. Neurons loaded with fura-2/AM were transferred to the stage of an inverted microscope (Nikon, Japan) in a perfusion chamber at room temperature. The inverted microscope was coupled with a dual-wavelength excitation spectrofluorometer (Intracellular imaging, USA). Cells were perfused with Krebs’ bicarbonate buffer (KB buffer, mM; 117 NaCl, 5 KCl, 1.2 MgSO_4_, 1.2 KH_2_PO_4_, 1.25 CaCl_2_, 25 NaHCO_3_, 11 glucose, pH 7.4). Drugs were added directly into the bathing solution during calcium measurement and the change in fluorescent intensity was monitored. For Fura-2 signal, the ratio of fluorescent signals obtained at 340 nm (F340) and 380 nm (F380) excitation wavelengths were recorded. F340/F380 ratio (R) was used to represent the [Ca^2+^]_i_ in the cells.

Neurons were also detected with confocal laser scanning microscopy (Olympus, USA) using fluo-4/AM as a calcium fluorescent indicator that could monitor real-time alterations of [Ca^2+^]^23^. The excitation and emission wavelengths of Fluo-4AM were 488 and 506 nm, respectively. All fluorescence measurements were made from subconfluent areas of the dishes so that individual neuron could be readily identified. All fluorescence measurements were made at room temperature. Image data were analyzed off-line.

### Electrophysiology

The cortical neurons were continuously perfused with oxygenated (95% O_2_ plus 5% CO_2_) artificial cerebrospinal fluid (ACSF; pH 7.4) containing (in mM): 125 NaCl, 3 KCl, 1.25 NaH_2_PO_4_, 2.4 CaCl_2_, 1.2 MgCl_2_, 26 NaHCO_3_, and 10 glucose. The GABAA receptor antagonist picrotoxin (PTX, 100 µM; Sigma) was present throughout the membrane excitability and mEPSC recording. Whole-cell recordings of neurons were made using a patch-clamp amplifier (Multiclamp700B; Axon Instruments, Burlingame, CA). Data acquisition and analysis were performed using a digitizer (DigiData1400A; Axon Instruments) and the analysis software Clampfit10.2 (Molecular Devices, Sunnyvale, CA), respectively. The resistance of patch pipettes was 4–7 MΩ. Two intracellular solutions were used, based on cesium methanesulfonate (for mEPSC recording) or K-gluconate (for membrane excitability recording) containing (in mM): 120 cesium methanesulfonate or 120 K-gluconate, 2 NaCl, 20 HEPES, 0.4 EGTA, 5 tetraethylammonium-Cl, 2.5 Na_2_ATP, and 0.3 GTP-Tris, pH 7.2–7.4 (adjusted by CsOH or KOH).

Membrane excitability experiments were elicited under the current-clamping conFigureureuration as described by Mu et al.^24^. The resting membrane potential was adjusted to -70 mV by injecting a small positive or negative current to make the recordings from different neurons comparable. A current-step protocol (from -25 to 70 pA, with a 5-pA increment and a 10-sec interpulse interval) was run to detect the rheobase current and number of evoked APs.

Miniature AMPAR-mediated EPSCs (mEPSCs) were collected (> 500 per cell) in the presence of 1 µM tetrodotoxin (TTX; Tocris.) under a voltage-clamped neuron at −70 mV. For each neuron, a random stretch of 150 mEPSCs was used to construct cumulative probability plots and to calculate mean mEPSC amplitude and frequency. NCX current was recorded according to the previous publication^25^. Briefly, neurons were voltage clamped at a holding potential of −70 mV up to a short-step depolarization at + 60 mV (60 ms). Then, a descending voltage ramp from + 60 to −120 mV was applied. The current recorded in the descending portion of the ramp (from + 60 to −120 mV) was used to plot the current–voltage (I–V) relation curve. The magnitudes of I_NCX_ were measured at the end of + 60 mV (reverse mode) and at the end of −120 mV (forward mode), respectively. To isolate I_NCX_, the same neurons of all experimental groups were recorded first for total currents and then for currents in the presence of Ni^2+^ (5 mM), a selective blocker of I_NCX_. To obtain the isolated I_NCX_, the Ni^2+^-insensitive unspecific currents were subtracted from the total currents (I_NCX_ = I_T_ − I_NiResistant_). External solution contained the following (in mM): 126 NaCl, 1.2 NaHPO_4_, 2.4 KCl, 2.4 CaCl_2_, 1.2 MgCl_2_, 10 glucose, and 18 NaHCO_3_ with 20 mM TEA, 50 nM TTX, and 10 mM nimodipine (pH 7.4). The pipette solution contained the following (in mM): 100 potassium gluconate, 10 TEA, 20 NaCl, 1 Mg-ATP, 0.1 CaCl_2_, 2 MgCl_2_, 0.75 EGTA, and 10 Hepes, adjusted to pH 7.2 with CsOH.

### Experimental design and statistical analysis

The data presented in this study reflect multiple independent experiments performed on separate days using different mice and contain more than three biological replicates. Values are presented as mean ± SEM and the statistical analysis was performed with GraphPad Prism 7, SPSS 21.0 and Clampfit. All sample sizes are indicated in the figure lengends. Two-way ANOVA was used when comparing two variables, one-way ANOVA was used for multiple group comparisons and student’s unpaired two-tailed *t* test was used when two groups were compared. Bonferroni’s multiple comparisons test was used for post hoc comparisons depending on the experiments. *p* < 0.05 was predetermined as the threshold for statistical significance.

## Results

### NKAα1^+/−^ mice are more susceptible to ischemic damage than NKAα1^+/+^ mice

As shown in Fig. 1a, the heterozygous NKAα1^+/−^ mice showed specifically a reduction in NKAα1 expression (*t* = 4.903, *p* = 0.0017), but not those of NKAα2 (*t* = 0.05783, *p* = 0.9555) and NKAα3 (*t* = 0.4842, *p* = 0.6430) in the cortical lysates of brain tissues, when compared to the NKAα1^+/+^ mice. Our results showed that total NKAα expression was also significantly reduced (*t* = 4.072, *p* = 0.0047). Consistently, the plasma membrane expression of NKAα1 (*t* = 3.671, *p* = 0.0104), but not those of α2 (*t* = 0.793, *p* = 0.4580) or α3 (*t* = 0.3526, *p* = 0.7364), was decreased significantly in NKAα1^+/−^ mice when compared to NKAα1^+/+^ mice (Fig. 1b). The infarct volume observed in NKAα1^+/−^ mice 24 h after tMCAO surgery was significantly larger when compared to that in NKAα1^+/+^ mice (Fig. 1c, *t* = 2.668, *p* = 0.0148). These data suggest that a reduced expression of NKAα1 protein, which is likely to be translated to a reduction in NKA activities in the brain may lead to increased susceptibility to ischemic injuries.

### Activation of NKAα by DR-Ab protected against ischemic damage

A polyclonal DR-Ab was raised in rats and purified with protein A/G (Fig. 1d, e). This DR-Ab can specifically bind to NKAα in membrane lysates of mouse brain (Fig. 1f), HEK293 and SH-SY5Y cells (Fig. 1g). Most importantly, a single dose pretreatment with DR-Ab (30 µg/mouse, i.c.v.) 1 h before tMCAO significantly reduced the infarct volume when compared to those treated with IgG purified from normal serum (Fig. 1h, *t* = 2.475, *p* = 0.0257). Moreover, Fig. 1i (Left) showed the penumbra area (P) and the corresponding area (CL) which were collected for NKA activity measurement. Sham group treated with NS showed no significant effect on NKA activity (Fig. 1i Middle). However, Fig. 1i (Right) showed that NKA activity in the penumbra area of tMCAO mice was significantly decreased when compared to corresponding area of sham-operated controls (*F* = 8.635, *p* = 0.0047, Bonferroni’s test, Sham vs. NS: p = 0.0136). This reduction was attenuated by DR-Ab pretreatment (Bonferroni’s test, NS vs. DR: *p* = 0.0090). When DR-Ab (30 µg/mouse, i.c.v.) was administrated as a posttreatment 1 h after reperfusion, similar reduction in infarct volume was observed (Fig. 1j, *t* = 2.3, *p* = 0.0336). These data strongly suggest that DR-Ab afford protection against ischemic injury by increasing NKA activities.

### DR-Ab protects neurons against glutamate-induced injury

**Fig. 2:**
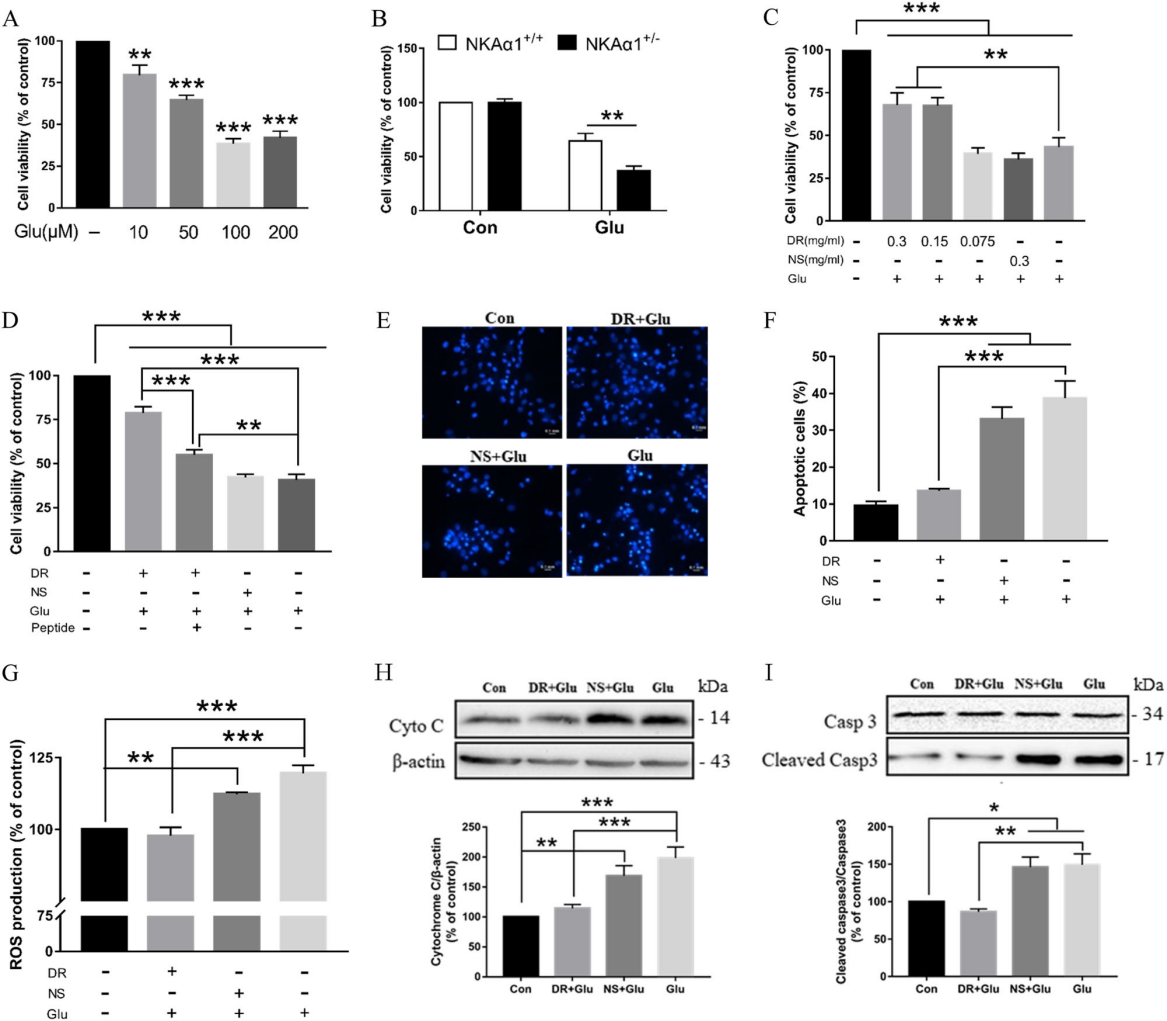
Protective effects of DR-Ab against excitotoxic injury in primary cultured cortical neurons. **a** Concentration-dependent effects of glutamate on cell viability. Cells were treated with glutamate + glycine (10:1) for 24 h, *n* = 6/group. One-way ANOVA followed by Bonferroni’s test, **p < 0.01, ***p < 0.001 vs control group. **b** Neurons from E17 NKAα1^+/−^ mice exhibited lower cell viability when compared to those from NKAα1^+/+^ mice when subjected to glutamate (50 µM) + glycine (5 μΜ) for 24 h, *n* = 6/group. Two-way ANOVA followed by Bonferroni’s test, **p < 0.01 with respect to NKAα1^+/+^ group. **c** Concentration-dependent protective effect of DR-Ab against glutamate-induced neuron injury. Cells were pretreated with DR-Ab (0.075, 0.15, 0.3 mg/ml) or IgG (0.3 mg/ml) purified from normal rat serum 1 h before glutamate (100 µM) + glycine (10 μΜ) stimulation for 24 h. *n* = 6/group. One-way ANOVA followed by Bonferroni’s test, **p < 0.01, ***p < 0.001. **d** Pretreatment with peptide (40 µM, 1 h) used to raise the DR-Ab significantly attenuated the protective effect of DR-Ab, *n* = 6/group. One-way ANOVA followed by Bonferroni’s test, **p < 0.01, ***p < 0.001. **e**, **f** Representative DAPI immunostaining images (**e**) and group data (**f**) showing that DR-Ab reduced cell apoptosis caused by glutamate, *n* = 6/group. One-way ANOVA followed by Bonferroni’s test, ***p < 0.001. **g** DR-Ab decreased ROS production stimulated by glutamate treatment for 30 min, n = 6/group. One-way ANOVA followed by Bonferroni’s test, **p < 0.01, ***p < 0.001. **h**, **i** Western blots showing that DR-Ab inhibited glutamate-induced cytochrome c release (**h**), *n* = 7/group, and caspase 3 cleavage (**i**), *n* = 6/group. One-way ANOVA followed by Bonferroni’s test, *p < 0.05, **p < 0.01, ***p < 0.001. DR: DR-Ab, Glu: glutamate (100 µM) + glycine (10 μΜ) for 24 h, NS: IgG purified from normal rat serum

Glutamate-mediated excitotoxicity is an important determinant of ischemic brain injuries following a stroke. Figure 2a shows that glutamate (10–200 µM, 24 h) concentration-dependently decreased cell viability in primary cortical neurons with a maximal reduction of ~ 65% at 100 µM (F = 47.5, *p* < 0.0001). When primary cultured cortical neurons from E17 NKAα1^+/−^ mice were compared to those from E17 NKAα1^+/+^ mice (Fig. 2b), it was found that the former were more susceptible to glutamate (glutamate 50 µM + glycine 5 μΜ, 24 h) toxicity than the latter (F(1,5) = 27.57, *p* = 0.0033, Bonferroni’s test, NKAα1^+/+^ Glu vs. NKAα1^+/−^ Glu: *p* = 0.0014). In addition, glutamate-induced toxicity was significantly attenuated by the pretreatment of DR-Ab (0.15 and 0.3 mg/ml, 1 h) (Fig. 2c, *F* = 27.65, *p* < 0.0001, Bonferroni’s test, Glu vs. Glu + DR 0.3 mg/ml: p = 0.0072, Glu vs. Glu+DR 0.15 mg/ml: *p* = 0.0083), while NS-treated did not show any beneficial effects (Bonferroni’s test, Glu vs. Glu+NS 0.3 mg/ml: *p* > 0.9999). As maximal effects were obtained at 100 μM glutamate and 0.15 mg/ml DR-Ab, these concentrations were used in all other experiments unless otherwise stated. Figure 2d shows that pretreatment with the DR peptide (DVEDSYGQQWTYEQR) at 40 µM, 1 h significantly attenuated the protective effects of DR-Ab (*F* = 91.13, *p* < 0.0001, Bonferroni’s test, Glu+DR vs. Glu+DR+peptide: *p* < 0.0001). This supports that such effect of the DR-Ab comes from binding to the NKA and presumably binding at the DR-region. Figure 2e shows that treatment with glutamate induced the appearance of condensed and fragmented nuclei, a characteristic of apoptosis. Pretreatment with DR-Ab abolished the glutamate-induced apoptosis in these cells as shown by the number of observed apoptotic cells (Fig. 2f, *F* = 23.93, *p* < 0.0001, Bonferroni’s test, Glu + DR vs. Glu: *p* < 0.0001). At the same time, DR-Ab blocked glutamate-induced ROS generation (Fig. 2g, *F* = 24.71, *p* < 0.0001, Bonferroni’s test, Glu + DR vs. Glu: *p* < 0.0001). Consistently, glutamate-induced cytochrome c release (Fig. 2h, *F* = 12.85, *p* < 0.0001, Bonferroni’s test, Glu + DR vs. Glu: *p* = 0.0005) and cleavage of caspase 3 (Fig. 2i, *F* = 9.795, *p* = 0.0003, Bonferroni’s test, Glu + DR vs. Glu: *p* = 0.0015) were also abolished. Taken together, these data strongly support the idea that by activating NKA, DR-Ab produced cytoprotective effects through inhibition of glutamate-induced ROS generation and apoptosis.

### DR-Ab inhibits glutamate-induced increased intrinsic excitability and synaptic transmission

**Fig. 3:**
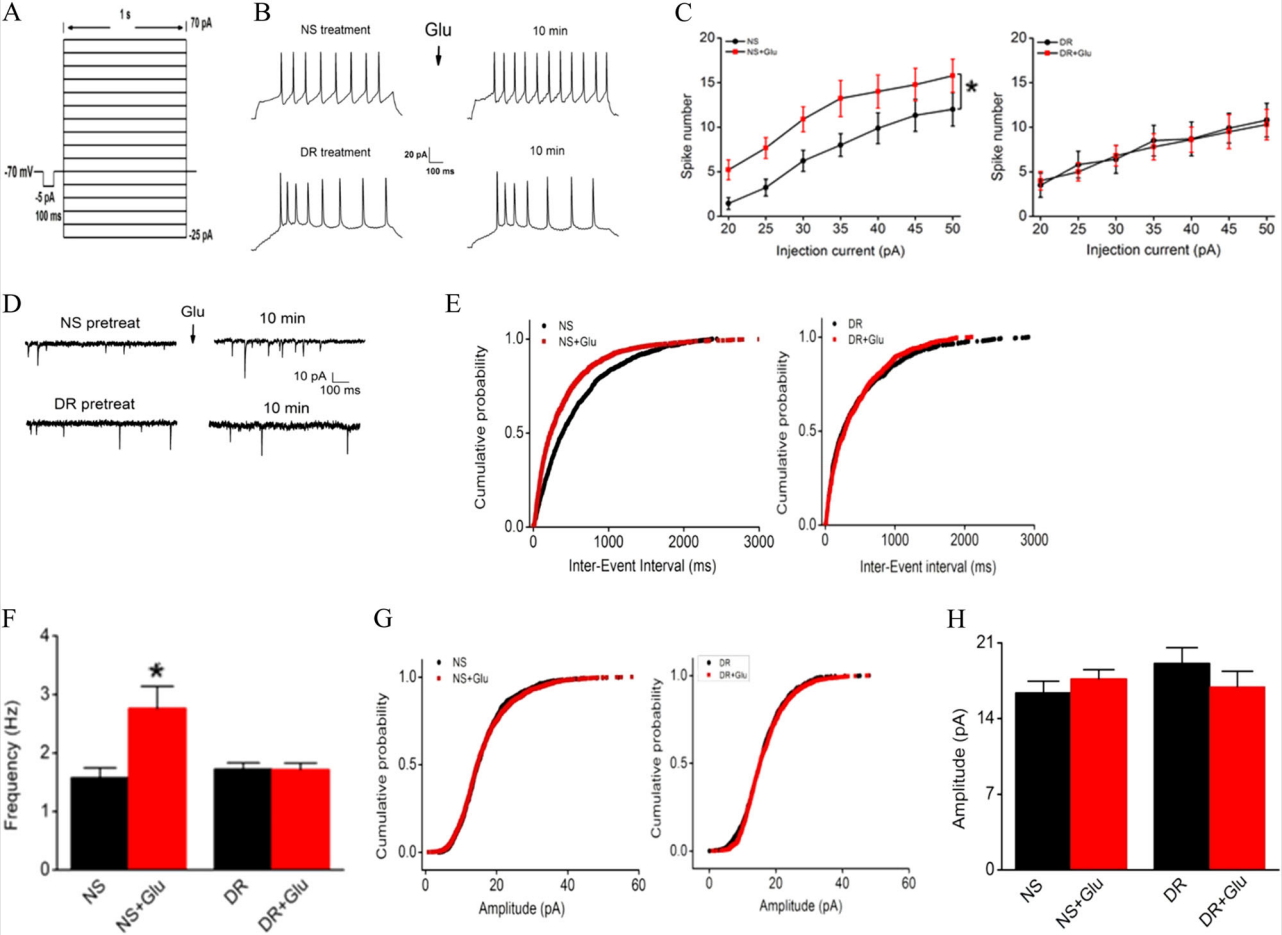
Effects of DR-Ab on glutamate-induced intrinsic membrane excitability and synaptic transmission in the primary cultured cortical neurons. **a** Pulse protocol showing that 35-pA current step from −25 to 70 pA were used to evoke action potential. **b** Representative APs in neurons before and after a brief application of glutamate in DR or NS pretreated neurons. **c** Group data showing that DR-Ab abolished the glutamate-induced increase in spike numbers. *n* = 9/group for left, *n* = 10/group for right. Two-way repeated ANOVA followed by Bonferroni’s test, *p < 0.05. DR: DR-Ab; Glu: glutamate (100 µM) + glycine (10 μΜ) for 30 s; NS: IgG purified from normal rat serum. **d–h** Representative tracings (**d**) and group data (**e-h**) showing a glutamate-induced an increase in mEPSC frequency (**f**), and left shift of the cumulative probability for mEPSC inter-event interval (**e**), but no effects were observed on the cumulative probability for mEPSC amplitude (**g**, **h**). DR-Ab abolished the effect of glutamate on mEPSC frequency (**e**, **f**). *n* = 8/group. **e** and **g**: Kolmogorov–Smirnov Test, **f** and **h**: Unpaired *t* test, two-tailed, *p < 0.05. DR: DR-Ab; Glu: glutamate (100 µM) + glycine (10 μΜ) for 30 s; NS: IgG purified from normal rat serum

Both synaptic input and neuronal intrinsic excitability are essential for functional neuronal output. In the present study, neuron excitability was determined using the current pulse protocol shown in Fig. 3a. Brief exposure to glutamate (100 μM, 30 s) would increase the number of spikes elicited by 35 pA injecting current step. Figure 3b shows that the number of evoked action potential (AP) was increased significantly upon glutamate treatment in the control (NS pretreatment) group, leading to a leftward shift of the current-spike (Fig. 3c left, Two-way repeated ANOVA, Bonferroni’s test, Interaction: current × group: F_6,96_ = 0.297, *p* = 0.937, Group: F_1,16_ = 4.86, *p* = 0.042). These effects were not observed in the DR-Ab pretreated group (Fig. 3c right, Two-way repeated ANOVA, Bonferroni’s test, Interaction: current × group: F_6,108_ = 0.231, *p* = 0.966, Group: F_1,18_ = 0.014, *p* = 0.908). Glutamate also significantly increased the frequency of mEPSCs (Fig. 3d–f. Figure 3e left: Kolmogorov–Smirnov Test, *p* < 0.0001; Fig. 3f: Unpaired *t* test, two-tailed, NS vs. NS + Glu, *t* = 2.8875, *p* = 0.0119), leading to a leftward shift of the cumulative probability distribution (Fig. 3e left), and pretreatment with DR-Ab again abolished these effects (Fig. 3d–f. Figure 3e right: Kolmogorov–Smirnov Test, *p* = 0.132; Fig. 3f: Unpaired *t* test, two-tailed, DR vs. DR + Glu, *t* = 0.0601, *p* = 0.9529). However, no difference was observed in the average amplitude of mEPSCs between the two groups (Fig. 3g, h. Figure 3g: Kolmogorov–Smirnov test, left, *p* = 0.4564, right, *p* = 0.1555; Fig. 3h: Unpaired *t* test, two-tailed, NS vs. NS + Glu, *t* = 0.9336, *p* = 0.3663, DR vs. DR + Glu, *t* = 1.0286, *p* = 0.3211). These data strongly suggest that DR-Ab is able to inhibit glutamate-mediated excitation.

### DR-Ab prevents glutamate-induced calcium overload

**Fig. 4:**
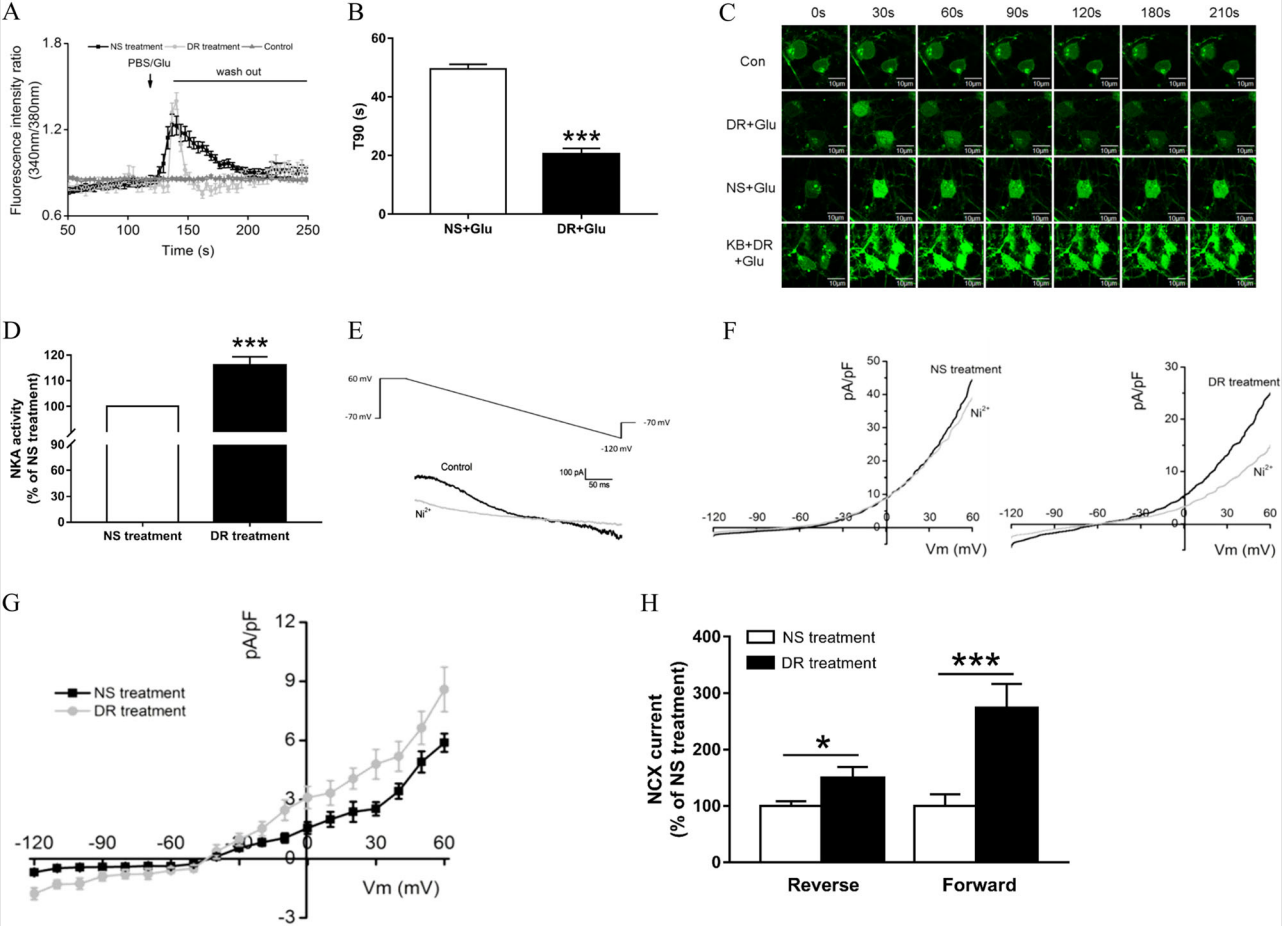
Effects of DR-Ab on glutamate-induced intracellular calcium increase and NKA and NCX activities in primary cultured neurons. **a** Pretreatment with DR-Ab for 1 h accelerated the extrusion of glutamate-induced increase in intracellular Ca^2+^ from neurons. **b** DR-Ab significantly reduced the decay time (T90, 90% of calcium level returned to baseline). Cells were treated with glutamate (100 µM) + glycine (10 µM) for 30 s followed by wash-out, *n* = 8 for NS + Glu group, *n* = 6 for DR + Glu group. Unpaired *t* test, two-tailed, ***p < 0.001. **c** Representative confocal microscopy images showing DR-Ab decreased intracellular calcium level in neurons treated with DR-Ab + glutamate. Blockade of NCX with KB-R7943 abolished the effect of DR-Ab. DR: DR-Ab; Glu: glutamate (100 µM) + glycine (10 μΜ) for 30 s; NS: IgG purified from normal rat serum. **d** DR-Ab stimulated NKA activity, n = 10/group. Unpaired *t* test, two-tailed, ***p < 0.001. **e** Typical membrane currents (lower panel) recorded from a neuron elicited by the ramp protocol (upper panel) in the presence of 5 mmol/L Ni^2+^. **f** Representative NCX currents recorded at different membrane potential in both NS (left panel) and DR-Ab (right panel) treatment groups. **g** Current–voltage (I–V) relation curve plotted from the currents recorded in both NS (*n* = 19) and DR-Ab treatment (*n* = 19) groups. **h** Statistic analysis showing that the effect of DR-Ab on NCX currents in both reverse and forward (outward) modes of operation, *n* = 19/group. Unpaired *t* test, two-tailed, *p < 0.05, ***p < 0.001. DR: DR-Ab; NS: IgG purified from normal rat serum

As shown in Fig. 4a, application of glutamate (100 μM in Mg^2+^-free solution containing 10 μM glycine) to the primary cultured neurons induced an increased intracellular calcium concentration through activation of glutamate receptors. Pretreatment of neurons with DR-Ab (DR + glu) attenuated the calcium overload induced by glutamate by accelerating the decay of intracellular Ca^2+^ concentration. The decay time from peak to 90% of baseline level was about 20 s in DR-Ab treatment group which was significantly shorter than that in the NS group (49.5 s) (Fig. 4b, *t* = 11.36, *p* < 0.0001). Time-lapse confocal microscopy analysis primary cultured cortical neurons treated with glutamate on DIV (days in vitro) 12 demonstrated an immediate rise in Fluo-4 fluorescence. Upon glutamate exposure, fluorescence intensity increased markedly within 30 s and remained at the high level over the whole recording period (210 s) (Fig. 4c, NS + glu treatment). Conversely, in the DR-Ab + Glu group, [Ca^2+^]_i_ was also increased but to a lesser extent and receded rapidly to a very low level by 90 s. An NCX inhibitor (KB-R7943, 100 μM, 30 min prior to DR-Ab) totally abolished this effect.

DR-Ab pretreatment significantly increased NKA activity (Fig. 4d, *t* = 4.8626, *p* < 0.0001) in these primary cultured cortical neurons. We also recorded NCX currents in both forward (outward) and reverse modes of operation with whole-cell patch-clamp technique. Figure 4e illustrates the I_NCX_ recording ramp protocol and the membrane currents from a neuron elicited in the presence and absence of 5 mmol/L Ni^2+^. The voltage-dependent I_NCX_ with or without DR-Ab treatment (Fig. 4f) and DR-Ab stimulated I_NCX_ at different voltage (Fig. 4g) were recorded. As shown in Fig. 4h, the results show that DR-Ab pretreatment significantly increased the NCX current by 49% in the reverse mode (*t* = 2.2908, *p* = 0.0279) and 174% in the forward mode (*t* = 3.7345, *p* < 0.0001). Together, these data confirm that DR-Ab effectively prevents Ca^2+^ overload during glutamate excitotoxicity. The mechanism may involve direct activation of NKA activities which, in turn, triggers an activation of NCX functions.

### DR-Ab pretreatment caused inhibition of GluR2 endocytosis

**Fig. 5:**
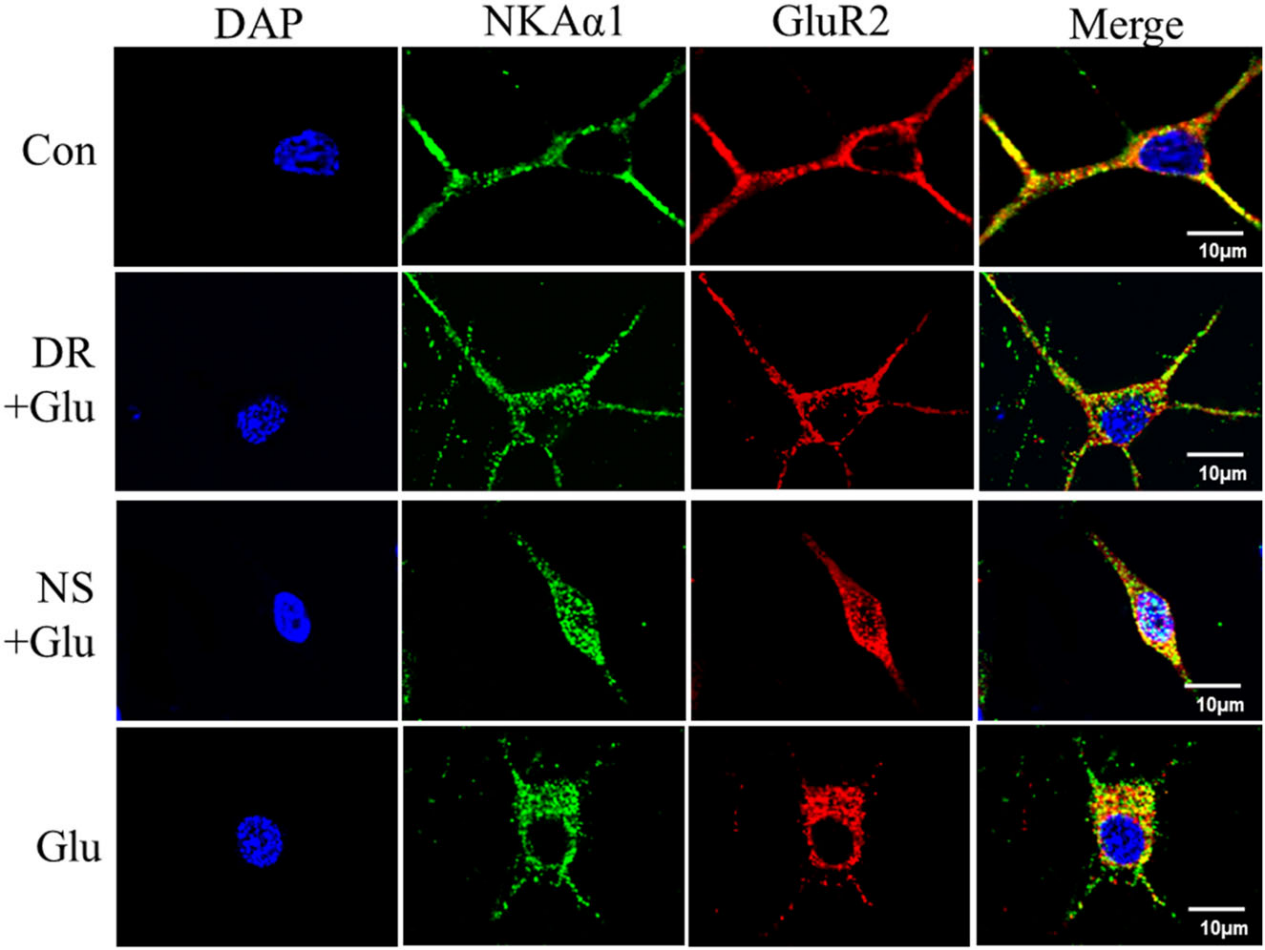
Confocal microscopy showing DR-Ab abolished the membrane loss of NKAα1 and GluR2 on the plasma membrane. Representative immunostaining images showing colocalization of NKAα1 and GluR2 on the plasma membrane (top panel), glutamate treatment induced membrane loss of NKAα1 and GluR2 through internalization (bottom panel), and DR-Ab pretreatment, but not control normal serum, attenuated the internalization of these 2 proteins. DR: DR-Ab; Glu: glutamate (100 µM) + glycine (10 μΜ) for 30 s; NS: IgG purified from normal rat serum

Constitutive and activity-dependent regulation of the AMPAR GluR2 content is recognized as an important mediator for both neuronal plasticity and vulnerability to excitotoxicity. To study whether DR-Ab can regulate GluR2 endocytosis, we first determined the colocalization of GluR2 and NKAα1 with confocal microscopy. We observed colocalization of NKAα1 and GluR2 in plasma membranes (Fig. 5 top panels). However, glutamate-induced internalization of both GluR2 and NKAα1. DR-Ab, but not the control antibodies purified from normal serum, markedly attenuated such internalization (Fig. 5).

**Fig. 6:**
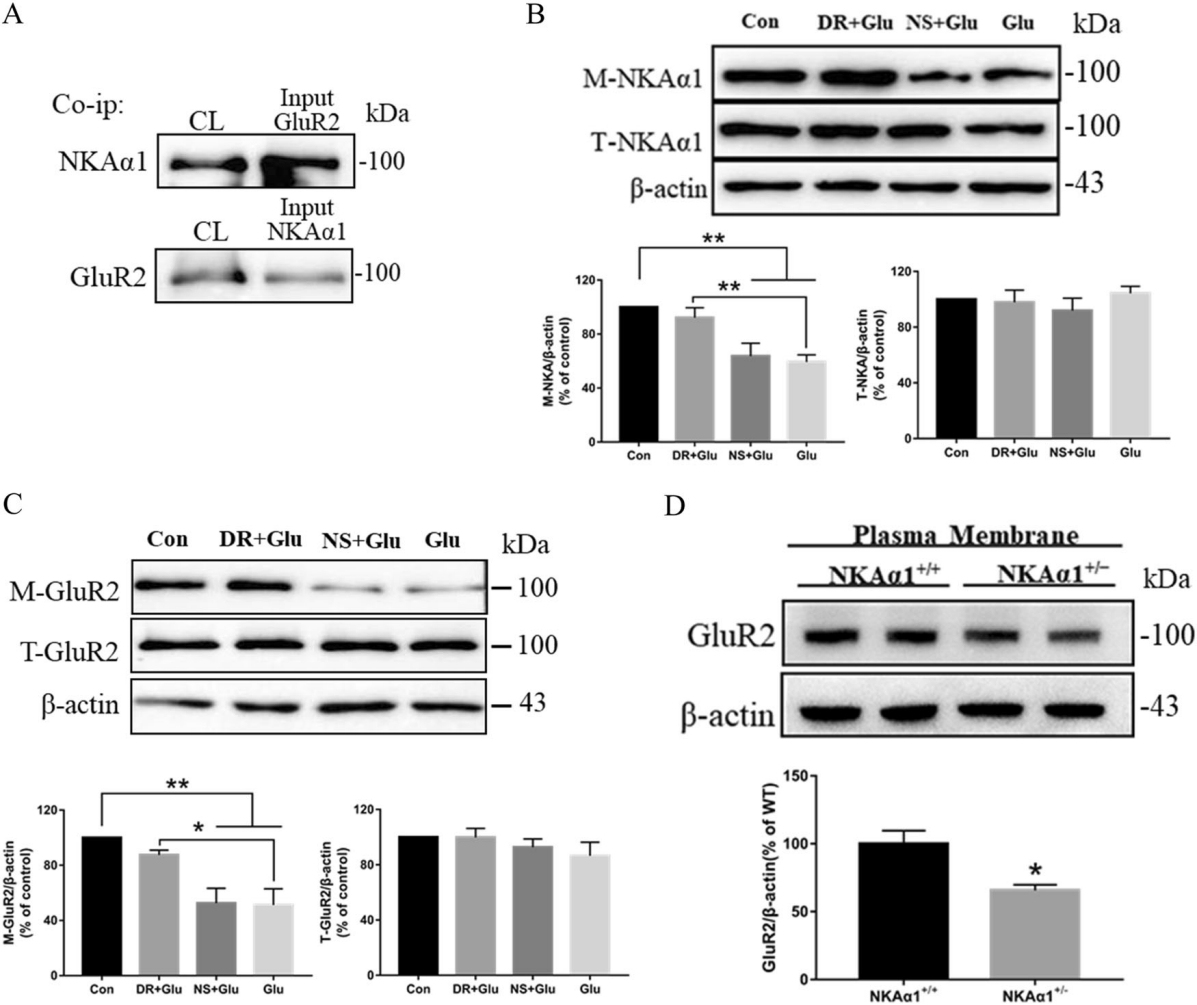
Effects of DR-Ab on the internalization of NKAα1 and GluR2. **a** Representative blots of co-immunoprecipitation assay showing direct interaction between GluR2 and NKAα1. Cell lysates were immunoprecipitated with anti-GluR2 and anti-NKAα1 antibodies and blotted with anti-NKAα1 and anti-GluR2 antibodies respectively. **b**, **c** Western blots analysis showing that DR-Ab reversed membrane loss of NKAα1 (**b**), *n* = 6/group, and GluR2 (**c**), *n* = 5/group, upon glutamate treatment. One-way ANOVA followed by Bonferroni’s test, *p < 0.05, **p < 0.01. DR: DR-Ab; Glu: glutamate (100 µM) + glycine (10 μΜ) for 30 min. NS: IgG purified from normal rat serum. **d** Western blots showing that the plasma membrane protein level of GluR2 was significantly reduced in NKAα1^+/−^ mice, *n* = 4/group. Unpaired *t* test, two-tailed, *p < 0.05 vs NKAα1^+/+^ mice. M-NKAα1: membrane NKAα1; T-NKAα1: total NKAα1

For confirmation, co-immunoprecipitation was performed to detect any direct association between NKAα1 and GluR2. As shown in Fig. 6a, significant amount of NKAα1 was detected in anti-GluR2 immunoprecipitated proteins (upper panel). Similarly, GluR2 can also be detected in anti-NKAα1 precipitated proteins (lower panel). Western blotting analysis showed that DR-Ab prevented the membrane loss of NKAα1 and GluR2 caused by glutamate treatment (Fig. 6b, c. Figure 6b, *F* = 9.718, p = 0.0004, Bonferroni’s test, M-NKAα1: Glu + DR vs. Glu: *p* = 0.0094; Fig. 6c, *F* = 9.176, *p* = 0.0009, Bonferroni’s test, M-GluR2: Glu + DR vs. Glu: *p* = 0.0312). As NKAα1^+/−^ mice exhibited reduced expression of NKAα1 (Fig. 1b), one might expect a reduction in the expression of GluR2 if there is such a direct association of these two proteins in the neuronal membrane. We therefore studied GluR2 protein expression in NKAα1^+/−^ mice and found that GluR2 expression was indeed significantly reduced in these mice when compared to the NKAα1^+/+^ mice (Fig. 6d, *t* = 3.274, *p* = 0.0169). These data suggest that the loss of membrane GluR2 may also lead to a reduction in membrane NKAα1 expression.

**Fig. 7:**
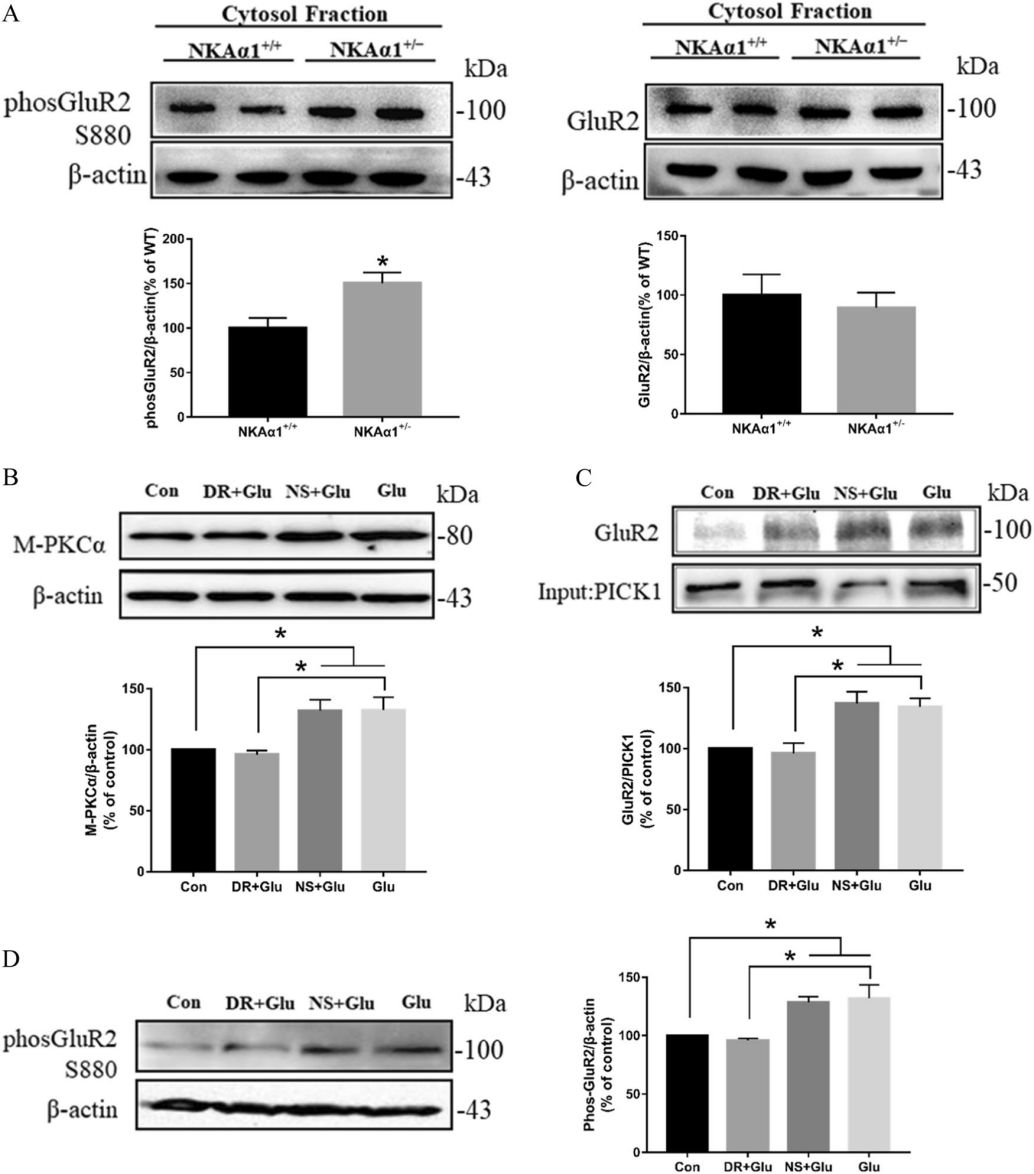
DR-Ab inhibited PICK1-mediated GluR2 phosphorylation. **a** Western blot showing increased GluR2 phosphorylation in NKAα1^+/−^ mice, *n* = 4/group. Unpaired *t* test, two-tailed, *p < 0.05 vs NKAα1^+/+^ mice. **b** Western blots analysis showing DR-Ab attenuated glutamate-induced PKCα translocation to membrane, *n* = 4/group. One-way ANOVA followed by Bonferroni’s test, *p < 0.05. M-PKCα: membrane PKCα. **c**, Co-immunoprecipitation assay showing DR-Ab reversed the interaction between GluR2 and PICK1 in neurons treated with glutamate, *n* = 4/group. One-way ANOVA followed by Bonferroni’s test, *p < 0.05. **d** Western blots analysis showing DR-Ab attenuated glutamate-induced GluR2 phosphorylation, *n* = 4/group. One-way ANOVA followed by Bonferroni’s test, *p < 0.05. DR: DR-Ab; Glu: glutamate (100 µM) + glycine (10 μΜ) for 30 min; NS: IgG purified from normal rat serum

As shown in Fig. 7a (Right panel), cytosolic GluR2, unlike membrane GluR2, was not decreased in NKAα1^+/−^ mice when compared to NKAα1^+/+^ mice (*t* = 0.4937, *p* = 0.6391). However, phosphorylated GluR2 was significantly increased (Fig. 7a left panel, *t* = 3.06, *p* = 0.0222). This may reflect increased internalization and phosphorylation of membrane GluR2 in these mice. As endocytosis of GluR2 is known to be preceded by PKCα-dependent serine 880 phosphorylation and interaction between PKCα and PICK1 are key to GluR2 phosphorylation^26^, we studied the changes in PKCα, PICK1, and GluR2 phosphorylation in cells treated with glutamate. Glutamate treatment induced PKCα translocation to membrane (Fig. 7b, *F* = 7.784, *p* = 0.0038, Bonferroni’s test, Con vs. Glu: *p* = 0.0350, Glu + DR vs. Glu: *p* = 0.0170), enhanced the association of GluR2 and PICK1 (Fig. 7c, *F* = 9.108, *p* = 0.0020, Bonferroni’s test, Con vs. Glu: *p* = 0.0289, Glu + DR vs. Glu: *p* = 0.0142) and induced GluR2 phosphorylation (Fig. 7d, *F* = 8.5969, *p* = 0.0026, Bonferroni’s test, Con vs. Glu: *p* = 0.0242, Glu + DR vs. Glu: *p* = 0.0110). These effects were all reversed by DR-Ab pretreatment. Therefore, it can be concluded that NKA activation by DR-Ab may inhibit GluR2 endocytosis by suppressing the PICK1/ PKCα pathway.

## Discussion

NKA activity is an essential part of normal neuronal functions in the brain. We observed, for the first time in this study, that genetic reduction of NKA activities in the form of NKAα1^+/−^ mice led to an increase in the brain infarct volume when these mice were subjected to tMCAO (Fig. 1). Conversely, DR-Ab, an antibody that binds to the DR region of NKAα1 and thus activates NKA function, was shown to afford neuroprotection against tMCAO-induced infarct volume (Fig. 1). As excitotoxicity due to the accumulation of extracellular glutamate^27^ is the major component in ischemic injuries, it was further confirmed that DR-Ab protected against glutamate-induced cell death in cultured neurons (Fig. 2). Calcium overload is a critical event in glutamate excitotoxicity involving both NMDAR and AMPAR. The Ca^2+^ influx can be counteracted by the action of Ca^2+^-ATPase or NCX operating in the forward mode. NCX can clear elevated Ca^2+^ more effectively due to its greater Ca^2+^ transport capacity^28^. Previous studies showed that NKA can functionally interact with NCX to control intracellular Ca^2+^ concentrations in the CNS and hence influence neurotransmitter release^29^ as well as prevent Ca^2+^ overload and neuronal apoptosis in excitotoxic stress^1^. Therefore, this may explain the observed neuroprotection afforded by DR-Ab following tMCAO. This assertion is supported by our observations that DR-Ab abolished glutamate-induced membrane excitation and synaptic transmission (Fig. 3). Moreover, DR-Ab effectively accelerated the decay rate of glutamate-induced calcium overload in these cells (Fig. 4). The fact that this effect of DR-Ab can be abolished by blocking NCX supports the idea that NKA is acting in concert with NCX. This is confirmed by patch-clamp recordings that DR-Ab markedly enhanced the NCX current by 3-fold in the forward mode as compared to an enhancement of only 49% in the reverse mode (Fig. 4).

NCX can be neuroprotective working in either forward or reverse mode. In the forward mode, NCX contributes to the lowering of Ca^2+^ overload and thus protecting neurons from Ca^2+^-induced neurotoxicity. NCX would be operating in a forward mode in the penumbral region where ATPase remains active^30^. The present results thus demonstrated that the observed neuroprotective effects of DR-Ab in the penumbral region can be, at least in part, attributed to NCX actions in the forward mode. Increased [Na^+^]i in the ischemic core due to NKA shutdown can also induce NCX to operate in the reverse mode. However, this can be beneficial for different reasons, firstly by promoting Ca^2+^ refilling into the ER through increased Ca^2+^ influx thus delaying ER stress; and secondly, by decreasing [Na^+^]i overload thus preventing necrotic cell death^25^.

NKA is known to co-localize with AMPAR at synapses and it influences AMPAR functions^31^. NKA dysfunction may induce a rapid reduction in AMPAR expression on the cell surface leading to a long-lasting depression in synaptic transmission perhaps due to increased degradation through proteasome-mediated proteolysis. The association of NKA and AMPAR appears to be mediated by NKAα1 subunits and the intracellular C-terminal of GluR2 subunits^3^. Our observations that NKAα1 and GluR2 colocalized in the plasma membrane (Fig. 5) and their association shown in immunoprecipitation experiments (Fig. 6) are entirely consistent with previous findings. Moreover, NKAα1^+/−^ mice with impaired NKA functions were also shown to exhibit reduced membrane GluR2 expression and increased phosphorylated GluR2 in the cytosol.

AMPAR contributes to the excitotoxic neuronal damage when GluR2 subunits are absent from the tetrameric receptor complex in pathological situations^26^. The absence of GluR2 in the AMPAR results in a switch from being Ca^2+^-impermeable to Ca^2+^-permeable^32^. In glutamate excitotoxicity, Ca^2+^-activated PKCα translocates from its constitutively cytosolic location to the plasma membrane by protein interacting with C kinase 1 (PICK1). Once bound to PICK1, PKCα phosphorylates GluR2 at Ser^880^. This allows the association of GluR2 with PICK1, and the subsequent internalization of GluR2^26^. By both confocal microscopy and Western blotting, we observed that glutamate treatment caused internalization of GluR2, indicating a probable switch of the AMPAR to being Ca^2+^-permeable (Figs. 5 and 6). Consistent to our observation that DR-Ab attenuated GluR2 internalization, we also observed activation of PICK1/PKCα pathway and increased GluR2 phosphorylation following glutamate treatment that were attenuated by DR-Ab (Fig. 7). This may be explained if the binding of DR-Ab at the DR region causes a stabilizing effect on the NKAα1-GluR2 complex and thus reduce GluR2 phosphorylation and internalization leading to a reduction in the conversion of AMPAR to the Ca^2+^-permeable form. More experiments are warranted to study how DR-Ab may stabilize the NKAα1-GluR2 complex, perhaps through conformational changes of NKA, and how such changes may prevent NKAα1 and GluR2 internalization.

**Fig. 8:**
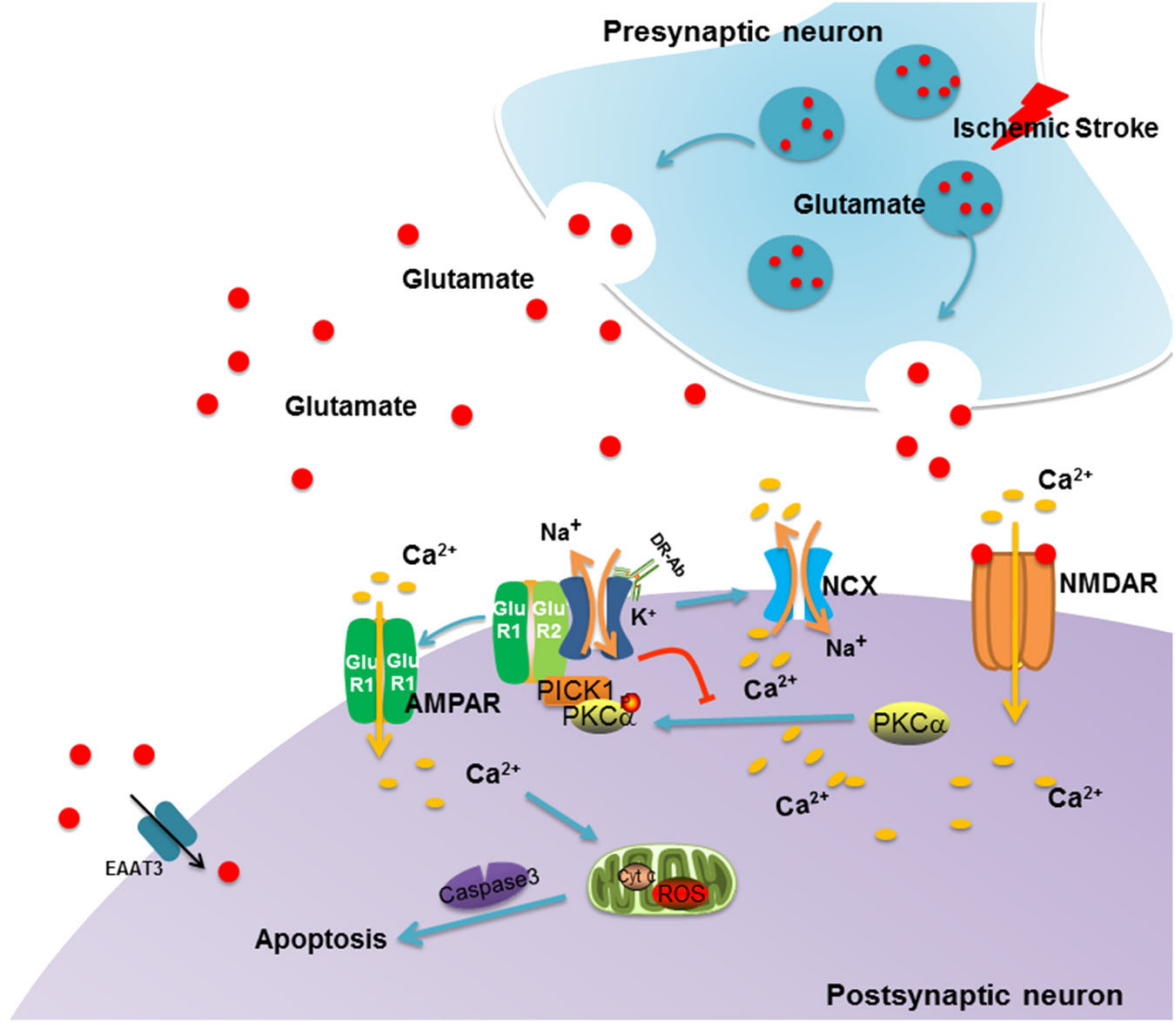
Schematic illustration showing the proposed mechanism of action of DR-Ab against glutamate-induced excitotoxicity. In ischemic stroke, increased glutamate release from presynaptic neurons into synaptic cleft activates AMPAR and NMDAR on the postsynaptic sites. Activation of NMDAR induces calcium influx which activates PKCα. The activated PKCα binds to PICK1, traffics to the membrane and phosphorylates GluR2 at Ser880. The phosphorylated GluR2 associates with PICK1 and internalizes from the cell surface. As a result, more calcium permeable GluR2-lacking AMPARs are expressed to exacerbate the intracellular calcium overload, which triggers the caspase 3 dependent neuronal death. By binding to the DR region of NKAα1, DR-Ab stabilizes the complex of NKAα1 and GluR2 on the plasma membrane. This reduces the calcium influx through AMPARs. DR-Ab also activates NKA which, in turn, stimulates NCX to extrude calcium

In summary, DR-Ab affords neuroprotection against ischemic injuries in vivo and also against glutamate excitotoxicity in cultured primary neurons. The mechanism involved is 2-fold: (1) DR-Ab activates NKA leading to a marked enhancement of NCX activities to extrude intracellular Ca^2+^. (2) DR-Ab stabilizes the NKAα1-GluR2 complex to reduce internalization of GluR2 and thus prevent the conversion of AMPAR from the Ca^2+^-impermeable form to the Ca^2+^-permeable form and thus reduce excitotoxic Ca^2+^ overload. The proposed mechanisms are presented in Fig. 8. The DR region of NKA is therefore a novel drug target for the development of peptidemimetics that simulate the actions of DR-Ab. These drugs may be of potential therapeutic value in the treatment of acute stroke.
